# Advances in Understanding the Genetic Mechanisms of Zebrafish Renal Multiciliated Cell Development

**DOI:** 10.3390/jdb11010001

**Published:** 2022-12-21

**Authors:** Hannah M. Wesselman, Thanh Khoa Nguyen, Joseph M. Chambers, Bridgette E. Drummond, Rebecca A. Wingert

**Affiliations:** Department of Biological Sciences, Center for Stem Cells and Regenerative Medicine, Center for Zebrafish Research, Boler-Parseghian Center for Rare and Neglected Diseases, Warren Center for Drug Discovery, University of Notre Dame, Notre Dame, IN 46556, USA

**Keywords:** multiciliated cell, development, ciliogenesis, Notch signaling, *mecom*, retinoic acid signaling, *etv5a*, *irx2a*, prostaglandin signaling, *ppargc1a*

## Abstract

Cilia are microtubule-based organelles that project from the cell surface. In humans and other vertebrates, possession of a single cilium structure enables an assortment of cellular processes ranging from mechanosensation to fluid propulsion and locomotion. Interestingly, cells can possess a single cilium or many more, where so-called multiciliated cells (MCCs) possess apical membrane complexes with several dozen or even hundreds of motile cilia that beat in a coordinated fashion. Development of MCCs is, therefore, integral to control fluid flow and/or cellular movement in various physiological processes. As such, MCC dysfunction is associated with numerous pathological states. Understanding MCC ontogeny can be used to address congenital birth defects as well as acquired disease conditions. Today, researchers used both in vitro and in vivo experimental models to address our knowledge gaps about MCC specification and differentiation. In this review, we summarize recent discoveries from our lab and others that have illuminated new insights regarding the genetic pathways that direct MCC ontogeny in the embryonic kidney using the power of the zebrafish animal model.

## 1. Introduction

**Figure 1:**
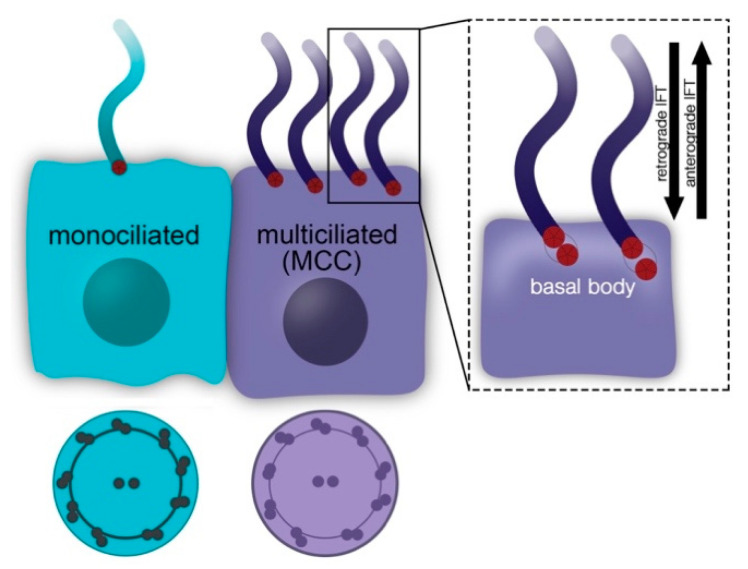
Comparison of the monociliated and multiciliated cell (MCC). Monociliated cells in an epithelial sheet possess a single cilium docked on the apical cell surface, which may be non-motile or motile, while the basal cell surface is adjacent to the basement membrane. By comparison, MCCs possess a multitude of cilia on their apical surface, and these exhibit coordinated movement that facilitates fluid propulsion. Each microtubule-based cilium is anchored by a basal body.

Cilia are microtubule-based organelles that protrude from cells, where they perform a tremendous variety of sensory and mechanical roles during normal tissue development and function. Nearly all vertebrate cells form a single non-motile primary cilium, which acts as a crucial regulator of signal transduction and cell behavior (Figure 1) [1,2]. Indeed, a growing list of complex genetic diseases and other syndromes are linked to primary cilium defects [3,4,5]. By comparison, some vertebrate cells become specialized to form multiple motile cilia, ranging from several to dozens or even hundreds in number, and are thus known as “multiciliated cells”, or MCCs (Figure 1) [6]. MCC formation requires a unique transcriptional program that orchestrates differentiation events, such as coordinated formation of basal bodies, which are microtubule-based organelles that are needed to support the microtubule-based cytoskeleton of each cilium, known as the axoneme (Figure 1) [7,8,9,10].

MCCs are amazing cells responsible for a plethora of essential physiological processes [11,12]. For example, they circulate cerebrospinal fluid in the brain and spinal cord, move the egg through the oviduct and fallopian tube, mobilize fluid in the efferent ducts to support spermatogenesis, and clear mucus in respiratory tract airways, where they provide a defense system against pathogens and debris [11,12]. In light of such key functions, it is not surprising that defects in MCC development and activity—such as frequency of beating or ciliary orientation—are now linked to childhood and adult pathologies that include hydrocephalus, infertility, chronic respiratory infections, and respiratory diseases such as cystic fibrosis [11,12].

Despite having central importance in human health, development of MCCs has remained historically understudied compared to that of other cell types over the last century. In part, this can be attributed to several experimental challenges in visualizing MCCs and assessing their behavior. More often than not, MCCs are located within organs nestled deep inside the vertebrate body plan, such as the aforementioned central nervous system, respiratory tract, and reproductive tracts. These anatomical locations proffer substantial challenges in monitoring MCC genesis and function within animals that possess very complex architectures and/or develop in utero, such as mammals.

In more recent years, however, research in eukaryotes ranging from protists to metazoans, the latter from the simplest invertebrates to the most complex vertebrates, has heralded a growing series of landmark advances in our understanding of MCC genesis [7,8,9,10,11,12]. For example, the embryonic amphibian epidermis has been a powerful tool to study MCC development and mucociliary biology [13,14,15]. Likewise, the planarian flatworm has been employed as a useful model to study epidermal MCC formation, where ciliary/MCC function can be readily monitored by observing each animal’s locomotion [16,17]. Further, the attributes of the zebrafish model have enabled rapid genetic assessment and identification of MCC regulators in several tissues, such as the olfactory pit, brain ventricles, and kidney [18,19,20,21]. Extensive research using mammalian cell lines, murine models, and human genetics has also unearthed powerful knowledge about MCC ontogeny in locales such as the respiratory system, brain, and reproductive tract [22,23,24,25,26,27,28].

Today, we now appreciate that the course of MCC development, also referred to by the term “multiciliogenesis”, is a multi-step process that begins with specification of the MCC fate in a precursor or progenitor cell [7,8,9,10,11,12]. This is followed by a suite of differentiation events that involve amplification of centrioles, which are then trafficked to the apical membrane where they will act as basal bodies—the structures that are used as the base for ciliary axoneme assembly. Finally, a cilia beating cycle is coordinated within each individual MCC and among neighboring MCCs to achieve a particular planar orientation and thus synchronized direction of fluid flow. Several contemporary reviews have provided a comprehensive discussion of topics such as the MCC transcriptional program, centriole biogenesis, basal body migration, and docking, as well as the fascinating mechanisms that establish polarized ciliary beating [7,8,9,10,11,12]. Here, we will discuss how our lab and others have leveraged the attributes of the zebrafish model system to implement developmental genetic approaches to elucidate novel insights about the molecular determinants of renal MCC fate choice and differentiation during kidney organogenesis.

## 2. The Zebrafish Pronephros Model of MCC Ontogeny

**Figure 2:**
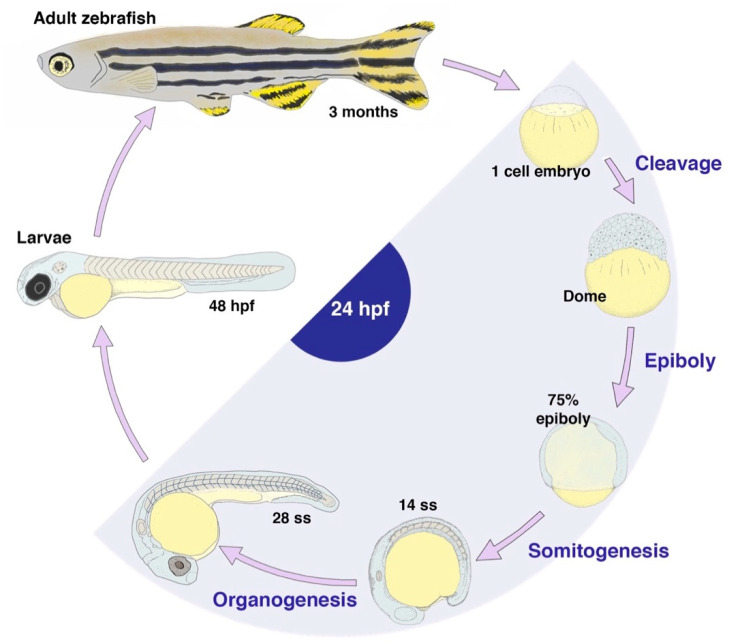
Zebrafish exhibit a rapid life cycle. Embryos are fertilized externally and progress through major developmental stages in the first 24 h post fertilization (hpf) (often denoted by the number of paraxial mesoderm sections, deemed as somite stages (ss)), forming a complete body plan with many major organs. Organogenesis continues through the 48 hpf time point and subsequent days as well, with larvae reaching sexual maturity at approximately 3 months of age.

The zebrafish, *Danio rerio*, is a particularly relevant animal model to elucidate the mechanisms of MCC development due to its high genetic conservation with humans. Approximately 70% of human genes have a zebrafish orthologue, and 82% of human disease-associated genes have a corresponding zebrafish orthologue—leading to popular use of this model in biomedical research over the past 30 years [29]. Zebrafish thrive in the aquarium setting and can be maintained well at a reasonable cost compared to mammalian research paradigms, such as the mouse [30]. Zebrafish reach sexual maturity in approximately three months and reproduce in large numbers: in the prime of their adult health, they can be mated weekly to generate single clutches of 100–250 embryos or more for experimental work [30]. Further, the embryological attributes of zebrafish, including their optical transparency and large size, allow for physical manipulation, such as microinjection and robust real-time visualization of morphogenic processes [30]. Zebrafish also develop ex utero and exhibit rapid development. After fertilization, early cleavage events begin almost immediately, reaching over 1000 cells by 3 h post fertilization (hpf). Gastrulation begins at approximately 5 hpf, with the first break in symmetry occurring at 6 hpf. Segmentation of the body plan and organogenesis begin at 10 hpf: structures such as the eye, heart, and kidney are well-formed by 24 hpf, and others, such as the liver, pancreas, and gastrointestinal tract, emerge a few days henceforth (Figure 2) [30,31]. These traits, combined with an advanced suite of molecular methods devised over time [X], enable powerful genetic approaches using the zebrafish model that can be implemented to systematically delineate the players in a developmental process such as MCC formation [32,33].

As mentioned in the introduction, zebrafish form MCCs in several locations [18,19]. One such site is the embryonic kidney, or pronephros [21]. In our lab, we study the pronephros to uncover fundamental principles of how renal progenitors undergo nephrogenesis—formation of the nephron structural and functional units of the kidney. The zebrafish pronephros consists of two parallel nephrons that arise along the trunk, just lateral to the midline [34]. These nephrons share a common blood filter at their rostral aspect, which is followed by a tubule that modifies the filtrate via reabsorption and secretion activities, and the tubules empty into collecting ducts that fuse with the cloaca for a single exit point [34]. This simple kidney anatomy is highly conducive for experimental work as its cellular components are readily accessible and thus easily visualized in both living and fixed samples [35,36]. In addition, the pronephros is among the most rapid organs to form in the zebrafish embryo [35,36]. It emerges from intermediate mesoderm-derived renal progenitors in just the first 24 h post fertilization (hpf) [35]. The renal progenitor cells transition from a mesenchymal to a polarized epithelial cell state, with clear apical and basolateral membrane distinctions between approximately 14 and 20 hpf [37,38], whilst commencing other differentiation events, such as ciliogenesis [39]. By 50 hpf, coordinated ciliary beating occurs in the pronephros, which is used to drive coordinated fluid flow toward the cloaca to accomplish waste excretion [39].

**Figure 3:**
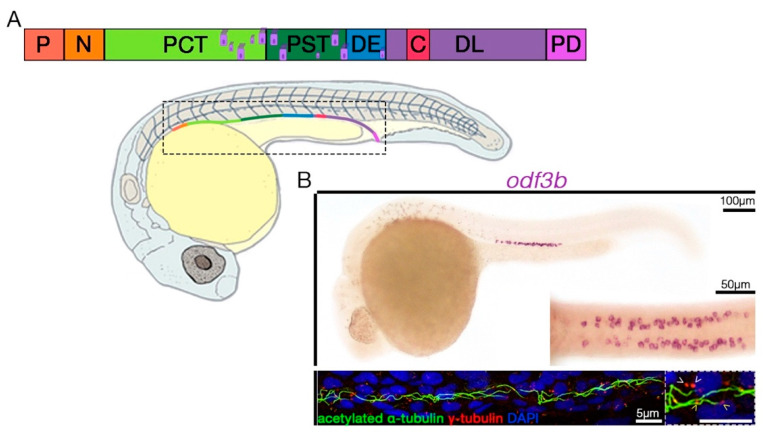
The zebrafish embryonic kidney contains a population of MCCs. (**A**) The nephron is comprised of several segments with specialized cell types (color coded), and MCCs are dispersed mainly in the proximal straight tubule (PST) region but also develop within the adjacent proximal convoluted tubule (PCT) and distal early (DE) segment. The schematic is representative of a 24 hpf (28 ss) animal. Other abbreviations: P, podocyte; N, neck; DL, distal early; CS, corpuscles of Stannius; PD, pronephric duct. (**B**) Whole-mount in situ hybridization to detect *odf3b* transcripts of a 24 hpf animal (**top**), which marks differentiating MCCs. Inset is a dorsal view of the pronephros, where individual MCCs are visible in both nephrons. Immunofluorescence (**bottom**) images of cilia (α-tubulin) and basal bodies (γ-tubulin) reveal both ciliated (white arrowheads) and unciliated (yellow arrowheads) basal bodies.

Each embryonic nephron in the zebrafish contains a population of between approximately 20 and 25 MCCs by the 24 hpf stage (Figure 3) [40,41,42]. This MCC contingent is easy to visualize, count, and track due to its small size [40,41,42]. The combination of fast development and a small but consistent MCC cohort makes the zebrafish pronephros a tractable model to delineate MCC genetic pathways—in particular, making it amenable to high-throughput reverse genetics and chemical screens for discovery and assessment of relevant factors [40,41,42].

Interestingly, the renal MCC population is intermingled within several nephron tubule segments, which are domains occupied by groups of specialized epithelial “transporter” cells (Figure 3) [43,44]. Upon their discovery, the MCCs were aptly described as being dispersed in a so-called “salt and pepper” fashion amongst the transporter cells [45,46]. It is useful to note that the transporter population has also been referred to in the literature as principal cells based on their expression of Na^+^, K^+^ ATPases as in their mammalian counterparts [45]. The MCCs have approximately 15–16 motile cilia that have a 9 + 2 microtubule structure and beat in a corkscrew along their longitudinal axis, propagating luminal fluid flow to drive excretion [39,45]. In contrast, the transporter cells possess a single cilium and function to secrete and recover specific solute molecules based upon their repertoire of solute transporter gene expression [39,47]. For example, the proximal straight tubule (PST) segment is comprised of cells that express *transient receptor potential cation channel, subfamily M, member 7* (*trpm7*), and *solute carrier family 13 member 1* (*slc13a1*), proteins involved in calcium and sodium/sulfate movement, respectively [47]. To date, researchers have identified four major tubular segments, which have analogous transcriptional profiles with mammalian nephron segments, the proximal convoluted tubule (PCT), aforementioned PST, distal early (DE), and distal late (DL) [47,48,49,50]. Of these, most MCCs form within the PST segment, with a few detected in parts of the flanking PCT and DE segments that are directly adjacent to the PST (Figure 3) [45,46,51].

**Figure 4:**
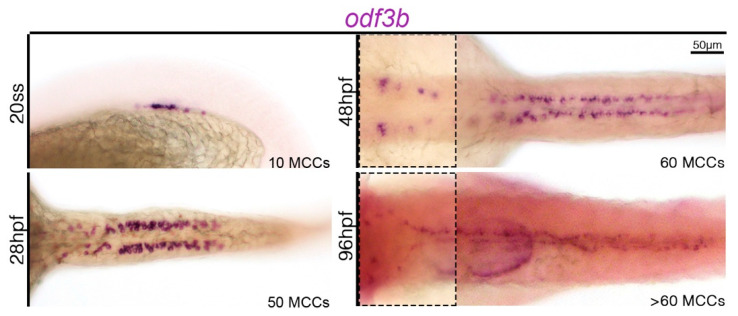
MCCs continue to develop throughout zebrafish organogenesis. Whole-mount in situ hybridization detects differentiating MCCs (marked by *odf3b*) at various stages. Approximately 10 MCCs arise at the 20 somite stage (ss) (**top**) and more than 60 can be detected by 96 h post fertilization (hpf) (**bottom**). Inset of 48 hpf and 96 hpf panels are from more rostral regions of the embryo. By the 96 hpf stage, the hook-like-shaped arrangement of renal MCCs is visible, suggesting that these MCCs occupy the proximal convoluted tubule.

Gene transcripts that mark MCC progenitors are detectable via whole-mount in situ hybridization as early as the 17–20 somite stage (ss) [45,46,51] (Figure 4). At this time, MCCs express such genes as ciliary transcription factor *rfx2* and *ctn4,* which encode a basal body protein and display several differentiated features by 24 hpf [v]. Localization of tubulin proteins revealed that the cilia are formed and anchored in basal bodies [45,46,51]. Nearly all basal bodies are ciliated [45,46,51] (Figure 4). Interestingly, although these two dozen or so MCCs are detected at 24 hpf, more MCCs emerge through the 36 and 48 hpf time points and beyond (Figure 4) [42,45,46,51]. Thus, continued MCC differentiation and maturation can be assessed over subsequent days. The source of these increased numbers requires additional study, and it has been hypothesized that increased proliferation in MCC bearing segments (PCT and DE) may contribute to their ontogeny [44]. As the pronephros begins to cleanse the circulation once morphogenesis of the glomerular filtration apparatus is completed at approximately 48 hpf, induction of sheer stress by fluid flow through the tubule results in rostral cell migration and distal segment proliferation [35,44,52]. Nonetheless, the structure and function of MCCs in the developing nephron have been well-characterized and provide a valuable model to elucidate the mechanisms that control their genesis.

## 3. The Role of Notch Signaling in MCC Fate Choice Is Highly Conserved

Seminal studies in developing zebrafish pronephros have shown that Notch signaling restricts MCC formation through its classical lateral inhibition mechanism [45,46]—a function that is conserved in other tissues where MCCs arise (e.g., frog epidermis, mammalian trachea) [6]. Notch receptors are transmembrane peptides that interact with Delta and Serrate/Jagged ligands on neighboring cells [53,54]. Upon ligand/receptor binding, cleavage by a γ secretase enzyme releases the Notch receptor intracellular domain (ICD) from the membrane, and the Notch^ICD^ translocates to the nucleus to activate transcription of target genes, such as Hes and HRT/HERP/Hey families of transcriptional repressors [53,54]. Abrogation of Notch signaling in renal progenitors, such as through loss of Jagged2a receptor activity, chemical treatment with γ secretase inhibitor DAPT (N-[N-(3,5-difluorophenacetyl)-L-alanyl]-S-phenylglycine t-butyl ester), or knockdown of either the Notch 1a or Notch 3 receptor, all lead to a significant increase in total MCC number [45,46]. Conversely, transgenic overexpression of Notch^ICD^ causes renal progenitors to adopt the transporter/principal cell fate at the expense of MCC fate selection [45,46]. Further, researchers identified *her9* as a critical downstream Notch target that participates in repressing expression of pro-cilia genes [45], such as *rfx2* [46], but also surmised that other not-yet-identified targets may also be involved [45].

**Figure 5:**
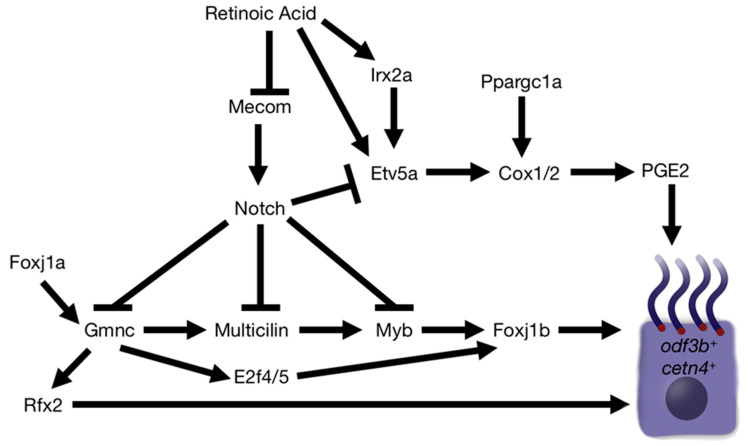
Working model of renal multiciliogenesis in the zebrafish embryo. Genes and signaling pathways demonstrated to be essential for MCC development are depicted.

Some positive regulators of MCC genesis downstream of Notch have been identified using the zebrafish pronephros model. These include Gmnc, Multicilin, Myb, and Foxj1, where Gmnc regulates MCC development by promoting Multicilin, while Myb and Foxj1 control differentiation steps, as in mammalian MCCs [7,8,9,10,11,12]. As the roles of these factors have been discussed very nicely in recent reviews [7,8,9,10,11,12], the following sections are focused on MCC fate and differentiation regulators that we and others have identified to be essential in renal MCC development. In these sections, we will discuss the findings that have led to an exciting emerging working model of renal multiciliogenesis that provides many opportunities for new hypotheses and future research (Figure 5).

## 4. Identification of Other Key Signaling Pathways and Transcriptional Components of the MCC Genetic Regulatory Network

### 4.1. Notch Is Positively Regulated by the Mecom Transcription Factor

In zebrafish pronephros, the transcription factor *mecom* restricts MCC fate upstream of Notch signaling [55]. Further, *mecom*-deficient embryos showed an increase in MCCs, similar to the effect of blocking Notch signaling [55]. Combined loss of *mecom* and Notch signaling did not show any further increase in MCC number [55]. As such, we hypothesized that *mecom* and Notch collaborate in the same pathway to limit MCC formation. To address this, we used transgenic line *Tg(hsp70:gal4; uas:notch1a-intra)* [56] to overexpress Notch^ICD1a^ and test whether expansion of MCC numbers in *mecom*-deficient embryos could be rescued with ectopic Notch signaling. Indeed, NICD activation by heat-shock in the absence of normal *mecom* expression resulted in fewer MCCs, consistent with the notion that Notch signaling acts downstream of *mecom* to restrict MCC fate [55].

### 4.2. Retinoic Acid (RA) Acts Upstream of Mecom Notch Signaling to Promote MCC Fate

RA is a vitamin A derivative essential for many developmental processes, including nephron segment patterning [47,48,49,57,58,59,60,61]. Interestingly, RA negatively regulates the domain of *mecom* expression in renal progenitors [47,49]. Given the roles of RA in regulating transporter cell identity, we hypothesized that RA might also modulate MCC fate choice and that it accomplishes this role partly through regulating *mecom*. Consistent with this, abrogation of RA biosynthesis with the inhibitor DEAB prevented MCC development, while exposure to elevated RA increased MCC numbers [55]. Additionally, RA mitigated these effects in part by inhibiting expression of *mecom* as MCC formation was partly rescued by *mecom* knockdown in DEAB-treated embryos [55].

### 4.3. Candidate Notch Targets: ETS Transcription Factors Etv5a/4

Several transcriptional regulators are requisite for MCC genesis in the zebrafish pronephros. Of these, Etv5a/4 are necessary to support MCC fate choice [58]. We initially hypothesized that Etv5a controls MCC fate because *etv5a* was co-expressed in renal precursors that express MCC marker *odf3b* [58]. Moreover, there was precedence from prior work implicating Etv5a in tissue patterning and ciliogenesis [62]. Knockdown studies, as well as over-expression of a dominant negative construct, revealed that *etv5a* is required for MCC formation in zebrafish pronephros [58]; *etv5a^sa16031^*+/− and *etv5a^sa1603^*−/− embryos also had reduced MCC numbers, confirming the knockdown findings and identifying a genetic model for further studies [51]. In each case, loss of *etv5a* led to a significantly reduced MCC contingent [51,58]. This number was further reduced in *etv5a*-deficient embryos that were deficient in the ubiquitously expressed, related family member *etv4* [58]. These results establish that Etv5a and Etv4 have redundant roles in MCC formation.

Interestingly, *etv5a* expression is negatively regulated by Notch signaling to partly constrain MCC number [58]. Given the central role of Notch in MCC fate, we examined if Notch interacts with *etv5a*. Notch signaling inhibits *etv5a* to restrict MCCs as DAPT treatment expanded the *etv5a* expression domain in the pronephros as well as increased MCC numbers [58]. In addition, *etv5a*-deficient embryos treated with DAPT had significantly fewer MCCs than DAPT treatment alone [58]. This indicates that Notch, either directly or indirectly, serves as a negative regulator of *etv5a*. Further, the relationship between Notch signaling, *etv5a,* and MCC formation was evaluated using the aforementioned transgenic line *Tg(hsp70:gal4; uas:notch1a-intra)* that expresses the Notch^ICD1a^ under temporal control mediated by heat-shock. In this context, there was a dramatic reduction in the length of the *etv5a* expression domain in NICD+ embryos and MCC number, further supporting the conclusion that Notch inhibits MCCs partly through affecting expression of *etv5a* [58].

### 4.4. The Iroquois (irx) Transcription Factor irx2a

The *Iroquois* family of transcription factors have been established as essential regulators of embryogenesis, specifically in the processes of patterning [63,64]. Of this family, *irx3b* and *irx1a* are expressed in PST-DE and DE regions of the zebrafish pronephros, respectively, and these factors are essential components of the gene regulatory network that regulates genesis of DE lineage [49,65,66,67,68,69]. Yet another member of this family, *irx2a*, is expressed in the PST-DE segments of the nephron and is essential for proper development of pronephric cell types, including MCCs [70]. Interestingly, *irx2a* colocalized with a subset of *odf3b^+^* cells at 24 hpf, but co-expression presented as a range in which some cells were independently *irx2a^+^* or *odf3b^+^* [70]. This modulation of *irx2a* expression suggests that perhaps *irx2a* marks MCC precursors, and the observed diminished expression is a result of MCC maturation [70]. This hypothesis was further supported as deficiency models of *irx2a* in the zebrafish resulted in a decreased number of MCCs as well as decreased expression domain of *etv5a* [70]. Changes in retinoic acid signaling also affected *irx2a* expression as treatment with exogenous RA expanded the *irx2a* domain and shifted it caudally, while inhibition with DEAB shifted the *irx2a* domain rostrally and caused it to be significantly decreased in length [70]. Currently, these data place *irx2a* downstream of RA and upstream of *etv5a* in the MCC regulatory pathway, yet additional studies are essential to determine the exact nature of the interactions amongst these regulators.

### 4.5. Prostaglandin Signaling Regulates MCC Specification and Differentiation

Several studies have illuminated important roles for prostaglandin signaling in MCC progenitor fate choice and subsequently in proper MCC differentiation. Prostaglandins (PGs, or prostanoids) are small lipid-derived molecules that regulate cellular activities in an autocrine or paracrine fashion. PGs are produced through several steps, beginning with phospholipases releasing arachidonic acid (AA) from membrane lipids. From here, AA is converted into prostaglandin intermediate PGH2 by cyclooxygenases [71]. There are two primary cyclooxygenases in vertebrates: COX-1, which is more common and functions to mediate the homeostatic functions of PGs, and COX-2, which is less common as it appears to be active only after being induced. Both COX enzymes are endoplasmic-reticulum- or nuclear-membrane-bound and function as homodimers with one catalytic and one regulatory subunit. The intermediate PGH2 is then further modified by specific synthases into one of the following PGs: PGE2, PGF2*α*, PGD2, or PGI2 [72]. Generally, this derivation of specific PGs occurs within the cell. However, it is possible for transcellular synthesis with COX and synthase activity to occur in other cells [72,73]. Additionally, prostanoids can diffuse or be transported out of the cell and into neighboring cells via diffusion or specialized transport proteins (ABCC4, MRP4, SLCO2A1), where they bind to their specific G protein-couple receptors (EP1-4, FP, DP and CRTH2, and IP, respectively) [74,75,76]. It is important to note that, in high enough quantities, it is also possible for prostanoids to bind non-specifically to other PG receptors [77]. However, bioactive PGs are usually found in low concentrations in vivo, in part due to their short half-lives, and bind to their specific receptors [72].

After PGs bind their respective receptors, they are involved in several biological processes, including G*α*-dependent signaling cascades (such as cAMP), MAPK, and PPAR signaling [72]. Proper balance of PG concentration is regulated not just by COX-initiated synthesis but also by degradation via 15-hydroxyprostaglandin dehydrogenase (12-PGDH) [71]. Importantly, PG receptors are found on many cell types. This explains the variety of cell types and corresponding functional effects associated with PG signaling [71]. Interestingly, PGE2 can be produced by many cell types and has been recognized to activate neutrophils, macrophages, and mast cells in inflammation while also being involved in fibroblasts and epithelial cells in other contexts [78,79,80,81,82].

Major inroads in understanding the developmental roles of prostanoids have been afforded through zebrafish-based research. Because zebrafish develop ex utero, this prevents maternal PGs from affecting embryonic development, unlike mammalian models where maternal contributions have prevented researchers from delineating PG requirements in embryogenesis [83]. Moreover, zebrafish Cox genes are very similar to their mammalian counterparts and are maternally deposited, further pointing to their importance in early development [83,84,85]. Overall, Cox activity has been noted as early as 3 hpf, prior to MCC specification in the pronephros and other tissues [86]. The essential components of prostaglandin signaling are also expressed in the developing pronephros, including receptors *ptger2a* and *ptger4a* [87] and Cox1 encoded by *ptgs1* [88,89,90]. Additional Cox enzymes, *ptgs2a* and *ptgs2b*, are expressed in the tissues immediately surrounding the pronephros (like the cloaca and somites), which could also serve as a source of prostanoids if excreted [88,89].

Prostaglandins have been linked to ciliary function for decades, including modulation of beat frequency in human airway cilia and other ciliated cells [91,92,93,94,95,96]. More recently, PGE2 was linked specifically to ciliogenesis as a mutation in the ABCC4 transporter in zebrafish resulted in hallmark ciliopathic phenotypes, such as body curvature, alterations in fluid homeostasis, and laterality defects [97]. ABCC4 localizes to the ciliary membrane of various cells, including the zebrafish Kupffer’s vesicle (KV), olfactory placode, and otic vesicle, as well as human retinal pigmentation epithelial 1 (hRPE1) cells and murine inner medullary collecting duct 3 (IMCD3) cells, and is essential for PGE2 signaling to drive intraflagellar transport (IFT) [97]. IFT is a highly regulated process driven by microtubule-based axoneme track and motor proteins, and its dysregulation often results in blunted or bulging cilia [98]. PGE2 specifically drives cAMP signaling, which, in turn, regulates anterograde IFT [97,99]. These findings have been applied to rescue cilia length in EP4-deficient cells and other ciliopathic models [90,97]. Additionally, prostaglandins have been recently proposed as a therapeutic for nephronophthisis as agonism of PG receptors rescues defective ciliogenesis [100].

In addition to cilia formation, PGE2 plays an important role in MCC fate choice. Cox1- and Cox2-deficient zebrafish embryos exhibit decreased numbers of pronephric MCC progenitors, marked by expression of Notch ligand *jag2b* and transcription factor *pax2a* at the 24 ss [51]. This decrease persists through at least 28 ss and is also associated with a decrease in the number of cells that express MCC differentiation marker *odf3b* [51]. Even though MCCs (*odf3b*^+^) are distributed along several segments (end of PCT, throughout PST, and DE), deficiency of Cox1/2 appeared to only affect MCCs in the proximal segments as the number of transporter cells increased at the expense of MCCs [51]. Even in the case that prostaglandin-deficient animals activated expression of mature MCC markers, they were not necessarily mature, as evidenced by the increase in the number of unciliated basal bodies [51]. Supplementation with dmPGE2 (a stable form of PGE2) could rescue MCC number in Cox1, Cox2, and double Cox1/2 deficiency, suggesting that PGE2 was indeed the major prostanoid of importance in the context of MCC genesis in the nephron [51]. These studies reveal that prostaglandin signaling, especially via PGE2, is essential for both MCC specification and cilia formation and maturation.

Furthermore, there is compelling evidence that prostaglandin signaling acts downstream of transcription factor *etv5a* during renal MCC development. This notion is supported by the finding that dmPGE2 supplementation partially rescues MCC number in the nephrons of *etv5a-*deficient zebrafish [51]. Interestingly, the proximal promoters of zebrafish *cox1* and *cox2* contain putative Etv5 binding sites [51]. In murine in vitro studies, Etv5 increases the transcriptional activity of the Cox2 promoter [101]. Taken together, this reasonably suggests a mechanism by which Etv5, or possibly a related family member such as Etv4, may directly regulate prostanoid biosynthesis to induce MCC fate choice. However, future studies are still needed to examine these possible molecular interactions in renal progenitors.

### 4.6. Modulation of Prostanoid Biosynthesis by ppargc1a

While prostaglandin signaling components have been well-characterized, the transcriptional network controlling this essential pathway is relatively understudied in multiciliogenesis and limited to the links with Etv5a discussed in the previous paragraph. This void has begun to fill with recent studies that identified *ppargc1a*, an essential coactivator of the PPAR pathway, as a key regulator of nephron formation [90,102,103]. Zebrafish deficient in *ppargc1a* exhibit many of the pleiotropic defects associated with defective cilia—body curvature, aberrant left–right symmetry, and pronephric cysts [90]. Consistent with these phenotypes, *ppargc1a* mutants had a decreased number of renal MCCs, and pronephric cilia of both multi- and mono-ciliated cells were shorter [90]. However, the number of basal bodies in each region of the nephron remained unaffected by *ppargc1a* deficiency, although there were fewer ciliated basal bodies [90]. These phenotypes (e.g., decreased MCC number and ciliated basal bodies) were strongly reminiscent of those observed in Cox deficiency models. Interestingly, *ppargc1a* deficiency also leads to decreased expression of *ptgs1* and endogenous levels of PGE2, and supplementation of either *ptgs1* transcripts or dmPGE2 was sufficient to rescue the ciliopathic phenotypes [90]. This suggests that prostaglandin signaling is under the regulatory control of *ppargc1a*. While the presence of putative PPAR binding sites upstream of the *ptgs1* open reading frame suggests that *ppargc1a* is likely acting in tandem with PPAR transcription factors, future experiments may look to interrogate the exact relationship between PPAR and prostaglandin signaling in the context of MCC genesis.

The *ppargc1a* deficiency phenotypes affect cilia formation of both MCCs and mono-ciliated transporter cells but push cells towards mono-ciliated cell fate. These two characteristics—cilia formation and MCC number—are not inextricably linked, as suggested by the unique phenotypes of IFT-specific-deficient animals. For example, knockdown of *ift88* results in decreased cilia length in the pronephros, while the number of MCCs remains constant [90]. While supplementation of either *ptgs1* transcripts or dmPGE2 can rescue the *ppargc1a* deficiency phenotypes, future studies are needed to parse out other gene regulatory network components that contribute to cilia outgrowth or MCC fate. Certainly, other factors of interest include but are not limited to the aforementioned *etv5a, irx2a, mecom,* and Notch signaling components. However, these factors are likely to be subsets of the regulatory network. Approaches to discover the missing players are one of the many future opportunities to build our understanding of these developmental events.

## 5. Swimming Ahead: Prospects and Challenges for Future Studies of MCC Development in Zebrafish

### 5.1. Expanding the Toolkit to Study Renal MCCs

As in other species, MCCs in zebrafish emerge at various developmental time points across different tissues and organs [18,19,20,21]. For example, committed pronephric MCC progenitors are detectable in situ as early as 20 ss, and their ciliated structures are discernable within a few hours, whereas analogous cells within the nasal placodes do not appear until after 48 hpf [18,19,20,21]. Interestingly, while we and others have observed a defined field of MCC progenitors in the pronephros tubule, the MCC number in the pronephros appears to increase over time, with MCCs emerging at more rostral locations (Figure 4) [42,51,58]. Thus, there are many questions remaining about the MCC lineage/MCC fate choice in the maturing pronephros. Proliferation does persist in the nephron after the segment pattern is initially established at 24 hpf, yet it remains unclear if MCC progenitors or less-specified renal progenitors are among this population. Indeed, adoption of MCC progenitor identity has been associated with exit from the cell cycle in other contexts [7,8,9,10,11,12].

One way to investigate these questions would entail creation of a reporter line that would mark the MCC progenitor identity in zebrafish. Transgenic reporter lines have been instrumental for in vivo time-lapse imaging and/or lineage tracing in developmental studies. Design of such lines, however, is not always straightforward. For example, previous work has established the *Tg(foxj1a:GFP)* line, with noted GFP fluorescence throughout the nephron [104,105]. Previous reports suggest that *foxj1a* is uniformly expressed in the nephron until 48 hpf, where it restricts to MCCs [106]. More recent studies, however, have found that *foxj1a* is not co-expressed with transporter marker *trpm7* at 24 hpf, suggesting that perhaps *foxj1a* restricts to MCCs earlier than 48 hpf or that some transporter cells may not have motile cilia [107]. The model suggesting principal cells in the zebrafish proximal pronephros are unciliated is counter to several other reports [45,46,51,90,106] but re-emphasizes the need for robust markers for MCC lineage tracing. Candidate markers for lineage tracing include MCC structural components such as *cent4*, *flr*, or *odf3b*. Further markers may be transcription factors, such as *rfx2* or master regulator *gmnc* [108,109,110]. Coupled with light sheet microscopy techniques, such transgenic lines would allow for tracking of MCCs in the nephron and other tissues to address if these cells migrate, proliferate, or perhaps even transdifferentiate. Understanding these key mechanisms is crucial to elucidating the origins of ciliated cells.

### 5.2. Assembling MCC Genetic Regulatory Network(s) and Connecting the Dot(s) across Species

The research discussed in the present work, as well as other important studies, have led to formulation of a working model for renal MCC development [107,108,109,110,111,112,113,114,115,116] (Figure 5). Future work is needed to identify the targets of transcription factors in this model, such as *mecom*, *irx2,* and *etv5a/4*. Chromatin immunoprecipitation approaches will be one powerful way to address this question. Additionally, there are several impressive datasets from investigations across metazoans that can be leveraged, e.g., [117,118,119]. These include lists of candidate genes and proteins, whose roles in multiciliogenesis are yet to be explored. Cross-species comparisons are bound to be useful given the high degree of conservation that appears to exist across mechanisms of MCC fate choice, differentiation, and ciliary development as well [7,8,9,10,11,12,15,21].

Given the importance of MCCs across tissues, questions remain concerning distinguishing mechanisms for MCC genesis in an organ-specific manner. For example, are transcription factors such as Mecom and Irx2a required for development of all MCCs, or just the pronephros? Several studies have pointed to the existence of tissue-specific programs as factors such as *mcidas* are expressed specifically in the pronephros but not in the ciliated nasal placode [110]. It is still unknown, however, if there are other “core” components required for lineage-specific tweaks. Indeed, in our own works, we often refer to “renal MCCs”. This may be an accurate and necessary handle to distinguish between unique MCC “types” across the body, or it may be a misnomer if pronephric MCCs are transcriptionally equivalent to MCCs of other tissues.

### 5.3. If and How MCCs Are Relevant to Human Kidney Disease States

While there are many fundamental similarities between nephrons across vertebrates, there are unique mechanisms required for progressive development of more complex kidney forms, such as mesonephros and metanephros [120,121]. The healthy adult human kidney does not contain MCCs, but they have been noted in the fetal kidney [122,123]. However, a number of clinical case reports have detected MCCs in renal biopsies from humans with several pathological states, e.g., [124,125,126,127,128]. These observations raise intriguing questions. Are these MCCs a contributing cause of the pathological state(s)? Are they a response to the disease, such as a mechanism that is responding to poor renal pressure and flow, and thus induced to promote flow? Further, how is understanding multiciliogenesis relevant to advancing our knowledge about congenital anomalies of the kidney and urinary tract (CAKUT)? [121]. We believe these topics are important for future study.

### 5.4. Do (Renal) MCCs Regenerate?

Finally, zebrafish provide an interesting opportunity to examine MCC genesis following tissue damage as they are a highly regenerative species. For example, embryonic nephrons [129,130,131] and the adult kidney can robustly repair injured epithelial cells. Further, the adult kidney can form de novo nephrons in a process aptly termed “neonephrogenesis” [132,133,134,135,136,137]. These abilities lead us to wonder if renal MCCs can be repaired after damage. Can renal MCCs regenerate if they are destroyed entirely, and how? Additionally, how might zebrafish MCC regeneration compare to mechanisms in other highly regenerative species, such as planarian, which have robust epidermal MCCs? Recent research has illuminated fascinating mechanisms of MCC removal during developmental tissue remodeling [138]. Further, researchers uncovered a phenomenon whereby terminally differentiated MCCs changed their identity to that of another cell type—fundamentally challenging the notion of terminal differentiation and opening many questions about MCC populations over time in other contexts [138]. These and similar studies will pave the way for understanding the dynamics of MCC populations during ontogeny as continued insights emerge about renal progenitor development [139,140], as well as adult life and disease across vertebrates [141]. Such topics are just more examples of exciting areas to investigate in the years to come.

## 6. Conclusions

The zebrafish model is an excellent model to study MCC development. The high degree of genetic conservation between teleost fish and humans makes it likely that they share fundamental mechanisms of MCC ontogeny. Understanding mechanisms of MCC progenitor fate choice in the kidney will reveal renal-specific insights and potentially mechanisms that are not cell-type-specific. It will be important to determine the identity of as yet obscure components of the MCC genetic regulatory networks and to undertake work to elucidate their roles. As so many aspects of MCCs remain enigmatic, there is an exciting future ahead for researchers working in this area of biology.

## Data Availability

Not applicable.
